# A Source of the Mysterious *m*/*z* 36 Ions Identified: Implications for the Stability of Water and Unusual Chemistry in Microdroplets

**DOI:** 10.1021/acscentsci.5c00306

**Published:** 2025-04-04

**Authors:** Casey J. Chen, Evan R. Williams

**Affiliations:** Department of Chemistry, University of California, Berkeley, California 94720 United States

## Abstract

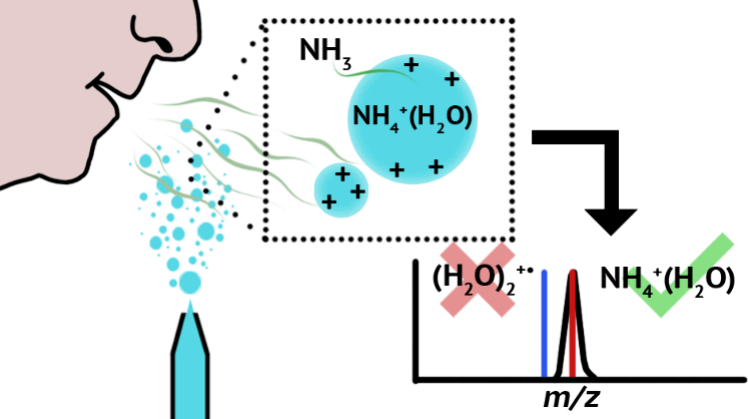

Many unusual reactions involving aqueous microdroplets have been reported, including nitrogen fixation at room temperature, production of abundant hydrogen peroxide, and formation of an ion at *m/z* 36, attributed to (H_2_O–OH_2_)^+•^, (H_3_O + OH)^+•^, or (H_2_O)_2_^+•^, which was used to support the hypothesis of spontaneous production of hydroxyl radicals. Here, *m*/*z* 36 ions and extensive hydrated clusters of this ion are formed using either nanoelectrospray ionization or a vibrating mesh nebulizer that produces water droplets ranging from ∼100 nm to ∼20 μm. Exhalation of a single breath near the droplets leads to a substantial increase in the abundance of this ion series, whereas purging the source with N_2_ gas leads to its near complete disappearance. Accurate mass measurements show that *m*/z 36 ions formed from pure water are NH_4_^+^(H_2_O) and not (H_2_O)_2_^+•^. Both the high sensitivity to trace levels of gaseous ammonia (unoptimized detection limit of low parts-per-billion) in these experiments and the likely misidentification of the *m*/*z* 36 ion in many previous experiments indicate that many results that have been used to support hypotheses about unusual chemistry and the effects of high intrinsic electric fields at microdroplet surfaces may require a more thorough evaluation.

## Abstract 2

Accurate mass measurements show that a *m*/*z* 36 ion that is sometimes formed from aqueous microdroplets is NH_4_^+^(H_2_O), not (H_2_O)_2_^+•^, and is abundant when droplets are exposed to human breath.

## Introduction

Aqueous microdroplets can lead to accelerated rates of reaction^1−6^ and to some unexpected chemistry.^7−15^ For example, evidence for ammonia formation from nitrogen in air at room temperature via a mechanism involving water microdroplets has been reported.^9,10^ Micromolar concentrations (0.3–30 μM) of hydrogen peroxide have been observed in some droplet experiments,^11−14^ and its formation has been thought to occur because of spontaneous production of abundant hydroxyl radicals at droplet surfaces.^15^ Formation of these species, which does not occur to a significant extent in bulk water, has been attributed to the high intrinsic electric field at the surface driving the breakup of water and formation of reactive species.^13,16,17^ In addition to the formation of hydrogen peroxide, evidence for this mechanism includes the formation of a *m*/*z* 36 ion that has been proposed to be (H_2_O–OH_2_)^+•^, (H_3_O + OH)^+•^, or (H_2_O)_2_^+•^,^7,15^ and the formation of abundant oxidation products of the radical scavengers of caffeine and melatonin.^15^ Spontaneous formation of such abundant reactive species in water droplets has important implications in environmental aerosol chemistry, human health, and chemical analysis by mass spectrometry using droplet ionization sources that is done in thousands of laboratories worldwide and motivates further investigations into their origins.

However, there is conflicting evidence that indicates that this hypothesis is incorrect and that water is stable at droplet surfaces.^18−21^ Recent results show that no detectable hydrogen peroxide is produced in droplets (<250 nM) when ozone is excluded from the environment,^19^ and <50 nM hydrogen peroxide is produced when dissolved oxygen is removed from water.^18^ When ozone was excluded in other experiments, hydrogen peroxide production decreased from 30 μM to as low as 0.3 μM, but the remaining hydrogen peroxide was attributed to formation directly from water due to the intrinsic electric field at the droplet surface.^13^ Pneumatic nebulization has been used to produce droplets in many of these experiments, and formation of reactive species have been observed, including ionized gases.^22−25^ These ionized gases could be formed by electrical discharge between droplets of opposite polarity that are generated in these sources.^21,22,24,26^ Recent results indicate that negatively charged droplets formed by either electrospray or pneumatic nebulization sources can lead to electronic excitation and ionization.^25^ As water evaporates from charged droplets, fission and charge emission can occur, but negatively charged droplets can also emit electrons. These electrons can be accelerated by nearby positively charged droplets or by external electrical fields, driving reactions with activation barriers in excess of 10 eV.^25^

Thus, a key to unlocking the mystery of these unusual reactions and the role of the intrinsic surface electric field, if any, is the identity and source of the *m*/*z* 36 ion that is produced in some aqueous droplet experiments^7,9,10,15^ but not in others,^9,21^ since this ion has been proposed as direct evidence for the formation of hydroxyl radicals at the surface of uncharged water droplets.^15^ This ion was not detected in nanometer sized droplets formed by nanoelectrospray nor was it detected from 2 to 20 μm diameter water microdroplets formed with a vibrating mesh nebulizer.^21^ Oxidation products of caffeine and melatonin were not reliably produced from water droplets formed by either nanoelectrospray or a vibrating mesh nebulizer, indicating that the source of these products in prior experiments^15^ was unrelated to the intrinsic surface potential at the droplet surface. A low abundance *m*/*z* 36 ion was observed with electrospray of water containing both FeCl_2_ and hydrogen peroxide, reagents known to produce hydroxyl radicals via Fenton chemistry, but accurate mass measurements were consistent with this ion being NH_4_^+^(H_2_O) and not (H_2_O)_2_^+•^. It was postulated that NH_4_^+^ could come from surface-pickup of ammonium salts used in native mass spectrometry and some HPLC separations, or it could originate from ammonia in human breath.^21^

(H_2_O)_2_^+•^ is not very stable, and it has been proposed that the inability to detect this ion in some experiments is due to energetic mass spectrometry source conditions.^27,28^ If this ion is indicative of hydroxyl radicals that are spontaneously formed at the surface of water droplets as a result of the intrinsic electric field, then its formation has significant implications in human health, aerosol chemistry, and electrospray mass spectrometry analysis owing to the presence of highly reactive species. Thus, the unambiguous identification and source of the ion at *m*/*z* 36 is critical to understanding how the intrinsic electric field at the surface of water droplets can drive reactive chemistries. Here, we demonstrate that no (H_2_O)_2_^+•^ is detected from droplets produced by electrospray or from a vibrating mesh nebulizer under soft mass spectrometer interface conditions, where large, weakly bound water clusters are transmitted and detected. Substantial ammonium water cluster formation can occur by exhalation of human breath near nanodrops formed by electrospray or micrometer sized droplets formed by a vibrating mesh nebulizer, and this can be a significant source of background *m*/*z* 36 ions. These results have important implications in the interpretation of prior data used to support the hypothesis that abundant hydroxyl radicals are produced at the surface of unactivated and even uncharged water droplets^15^ and for the production of ammonia from nitrogen gas with room temperature microdroplets.^9,10^

## Results and Discussion

**Figure 1:**
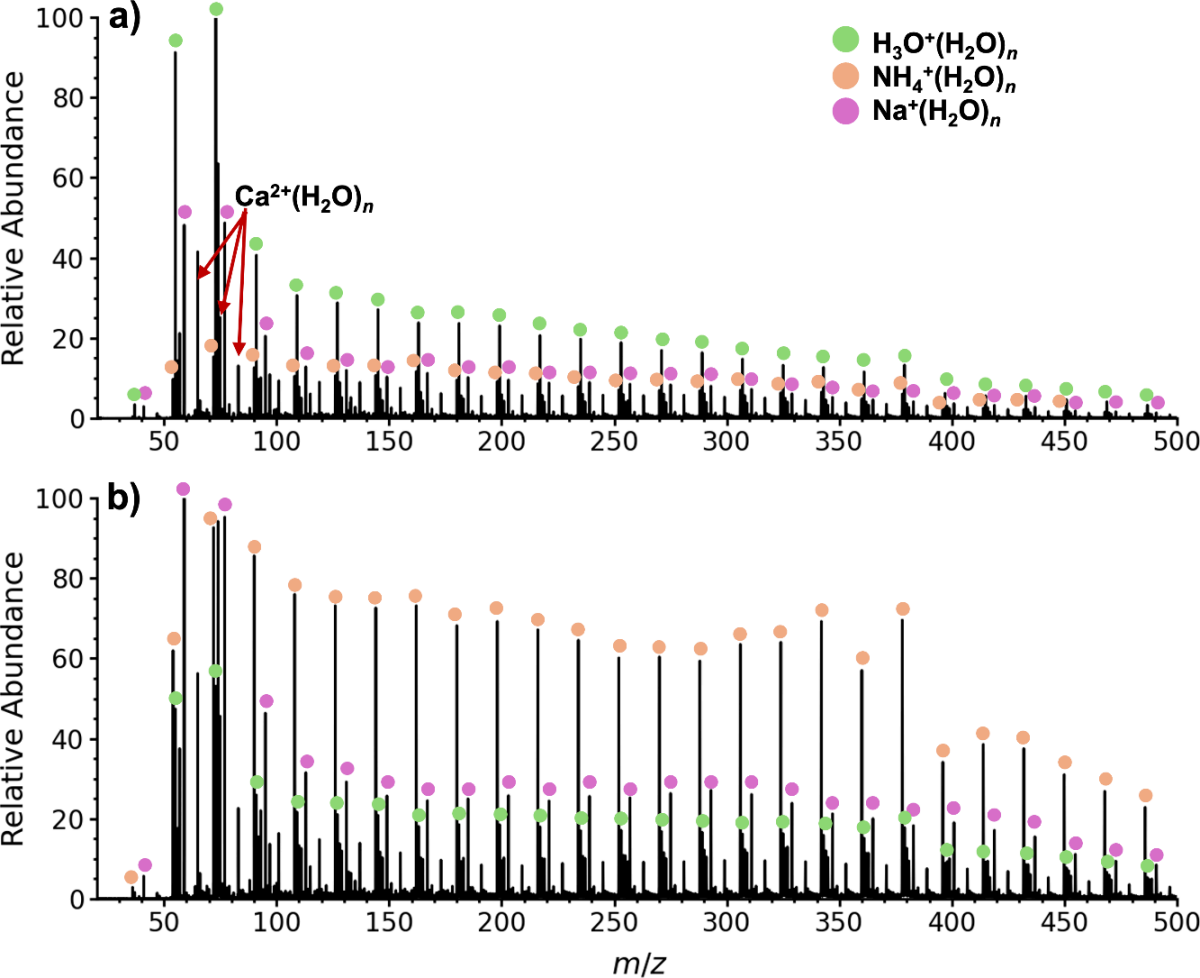
Nanoelectrospray mass spectra of 1.0 μM aqueous acetic acid solution using a partially enclosed ion source (Figure S7) in a) ambient laboratory air and b) summed for ∼12 s during which an individual exhaled a single breath ∼12 in. from the instrument source housing. The total ion current during these data acquisitions is shown in Figure S2.

Initial experiments were performed with nanoelectrospray of a 1.0 μM aqueous acetic acid solution to tune conditions of a Waters QTOF Premier mass spectrometer to preserve large, protonated water clusters that are produced by spraying water. (H_2_O)_2_^+•^ is weakly bound and it has been hypothesized that this species may not be observed in some experiments with water droplets because it has low stability.^15,27,28^ A nanoelectrospray mass spectrum of acidified but otherwise pure water solution obtained under soft instrument conditions that reduce the effects of ion heating is shown in Figure 1a. The most abundant ions correspond to a broad series of H_3_O^+^(H_2_O)_*n*_ clusters, *n* = 1–53, with less abundant Na^+^(H_2_O)_*x*_, K^+^(H_2_O)_*y*_, Ca^2+^(H_2_O)_*z*_, clusters also formed. Sodium, potassium and calcium are commonly associated with glassware, such as the borosilicate electrospray emitters and glass syringes used to load solution into these emitters. The large protonated water clusters are weakly bound,^29^ and their presence indicates that the source and instrument conditions are sufficiently soft that these weakly bound clusters remain intact and are readily detected. La^3+^(H_2_O)_*17–67*_ are formed from an aqueous LaCl_3_ solution under these same conditions (Figure S1). La^3+^(H_2_O)_*n*_ requires a minimum of 17 water molecules to be stable in the gas phase,^30^ indicating that the water clusters formed in these experiments are preserved throughout the entire mass spectrometry analysis. Similar soft conditions are used to directly mass analyze intact 20+ nm diameter water nanodrops formed using nanoelectrospray with charge detection mass spectrometry.^31,32^ The preservation of large water clusters in our experiments indicates that these conditions are less activating than those that have been used previously, where (H_2_O)_2_^+•^ detection was reported. Thus, if this ion was formed, it would not be expected to dissociate in this analysis due to excess energy (although we expect that it would be highly reactive).

There is a low abundance ion at nominal *m*/*z* 36 along with higher mass ions separated by 18 Da, consistent with larger water clusters associated with this ion (Figure 1a). Results from exhaling a single breath ∼12 in. from the source housing of the mass spectrometer leads to a fast rise in the total ion chromatogram (Figure S2) that lasts approximately 12 s before returning close to baseline. Results for the summed spectra from this 12 s period are shown in Figure 1b. The abundances of the *m*/*z* 36 ion and higher-order water clusters of this ion that are separated by 18 Da increase significantly relative to the signal for protonated water clusters and are the dominant ion series in this spectrum. This ion series in the mass spectrum acquired without exhaling near the instrument has a summed abundance that is ∼37% that for the protonated water clusters (Figure 1a, *n* = 1 – 25), but this value increases to ∼300% from the single exhalation of breath near the instrument (Figure 1b and Table S1). Exhaling breath at the entrance to the housing that encloses the electrospray emitter leads to an even greater abundance of this series, where it is 440% the abundance of the protonated water clusters (Figure S3). These results provide evidence that the ion series separated by 18 Da and starting at *m*/*z* 36 may be related to exposure of the charged droplets to human breath. Extractive electrospray has been used to measure organic compounds exhaled in human breath with high sensitivity,^33^ so this result is not unprecedented. Although the *m*/*z* 36 ion also appears at very low abundance without exhaling near the instrument, the instrument operator was located at the data acquisition system that is adjacent to the instrument in a standard orientation and the operator continued to breath normally throughout these experiments.

**Figure 2:**
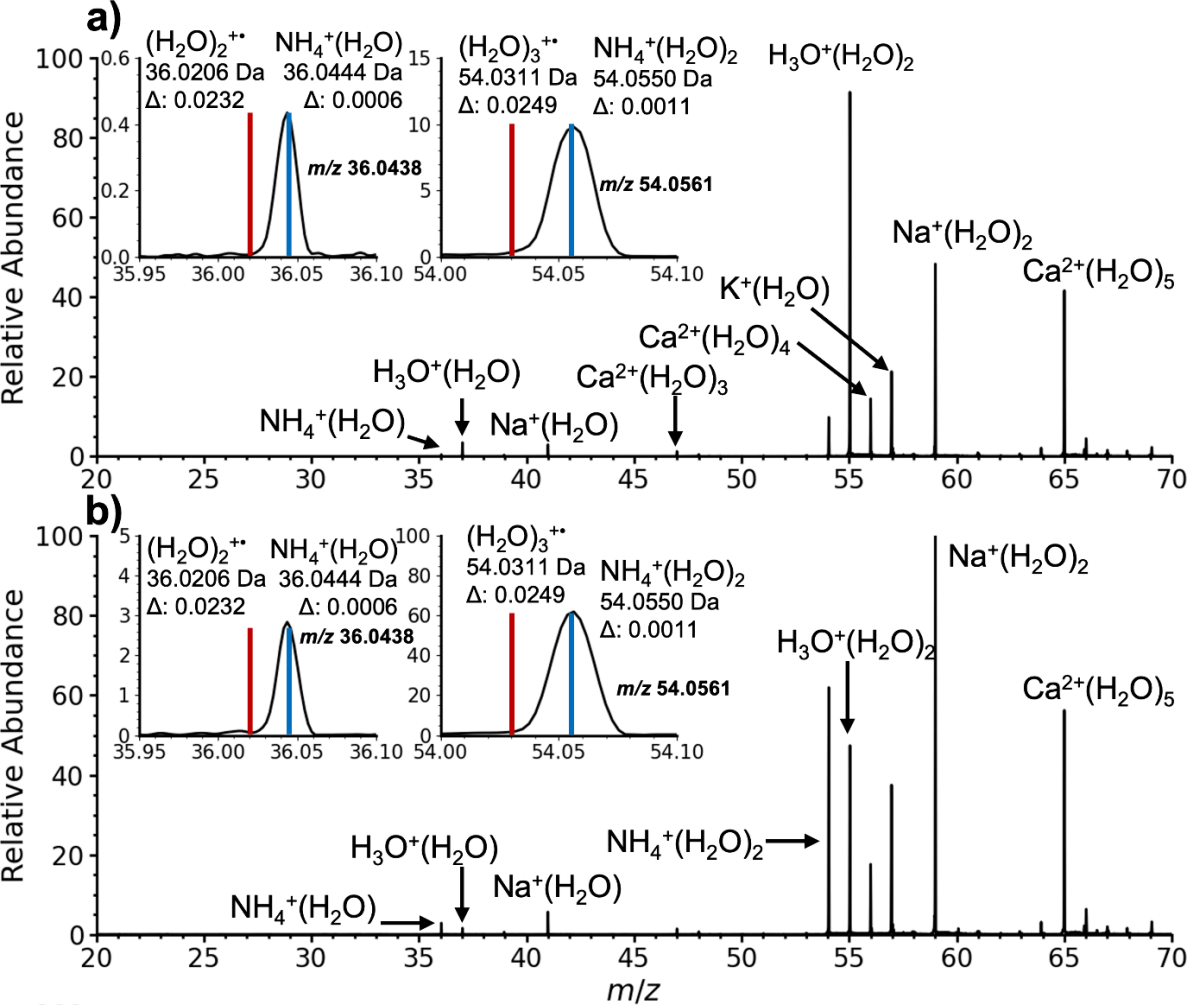
Expansion of data shown in Figure 1 around the region of *m/*z 20–70 from nanoelectrospray of a 1.0 μM aqueous acetic acid solution in a) ambient laboratory air and b) summed for 12 s during which an individual exhaled a single breath ∼12 in. from the instrument source housing. Insets are expansions around *m*/*z* 36 and *m*/*z* 54 showing accurate mass measurements of these ions and the exact masses of NH_4_^+^(H_2_O) and NH_4_^+^(H_2_O)_2_ (blue lines) and (H_2_O)_2_^+•^ and (H_2_O)_3_^+•^ (red lines).

NH_4_^+^(H_2_O) and (H_2_O)_2_^+•^ have the same nominal mass but different exact masses (36.0444 and 36.0206 Da, respectively). The instrument resolution of ∼1900 and mass accuracy is sufficient to differentiate these two species. An expanded mass range around *m*/*z* 36 from the Figure 1 data is shown in Figure 2. The measured *m*/*z* is 36.0438. This mass is consistent with that of NH_4_^+^(H_2_O) (blue line in Figure 2 insets; Δ*m* = 0.0006 Da) and not (H_2_O)_2_^+•^ (red line in Figure 2; Δ*m* = 0.0232 Da). The measured *m*/*z* of H_3_O^+^(H_2_O) and Na^+^(H_2_O), ions that are not used in either the external or internal calibration, are 37.0274 (Δ*m* = 0.0010 Da) and 40.9996 (Δ*m* = 0.0001 Da), respectively, indicating the level of mass accuracy achieved. The ion at *m*/*z* 54.0561 in Figure 2 is consistent with NH_4_^+^(H_2_O)_2_ (Δ*m* = 0.0011 Da) and not (H_2_O)_3_^+•^ (Δ*m* = 0.0250 Da), indicating that this ion is also part of the NH_4_^+^(H_2_O)_*n*_ cluster series. Table S2 provides the exact mass measurements of these and other ions. Other potential elemental compositions of the *m*/*z* 36 ion, including C_3_^+•^ and SH_4_^+•^, are ruled out based on mass deviation (Δ*m* ≥ 0.0410; Table S3), making the identification of the *m*/*z* 36 ion as NH_4_^+^(H_2_O) unambiguous.

Many different volatile organic compounds are in exhaled breath.^34^ The concentration of ammonia is around a few hundred ppb, but there can be large differences between individuals that depend on several factors, including diet.^35,36^ One study of a normal healthy population of 30 individuals showed that ammonia concentrations ranged from 29 to 688 ppb with an average value of 265 ppb.^35^ Elevated ammonia levels in breath have been linked to complications associated with the liver, kidneys, and stomach. In a separate experiment, some variation in the abundance of the *m*/*z* 36 ion and clusters separated by 18 Da occurred when four different healthy individuals exhaled near the instrument, but this variation was less than a factor of ∼4 (Figure S4).

An important question to address is why the signal in the mass spectrometer is so sensitive to ammonia in breath despite its relatively low concentration compared to water and some other volatile compounds. Ammonia has a significantly higher propensity to charge than does water, with a lower ionization energy (10.07 eV compared to 12.62 eV for H_2_O) and a significantly higher gas-phase basicity (819.0 kJ/mol compared to 660 kJ/mol for H_2_O).^37^ Thus, the transfer of a proton from protonated water to ammonia is highly exothermic. Water dimer is slightly less basic (∼800 kJ/mol),^38^ but larger water clusters should be more basic than water dimers and may not as readily proton transfer directly to gaseous ammonia. Rather, gaseous ammonia is likely incorporated into the droplets or protonated water clusters, and smaller clusters of protonated ammonia are made through the loss of neutral water molecules from larger clusters or nanodrops. A clathrate cage of water molecules occurs for NH_4_^+^(H_2_O)_20_,^39,40^ indicating the high stability of ammonium in water clusters. The greater abundance of the ion at *m*/*z* 378 (the mass of NH_4_^+^(H_2_O)_20_) compared with adjacent ions in this series provides additional evidence that these water clusters contain ammonium.

**Figure 3:**
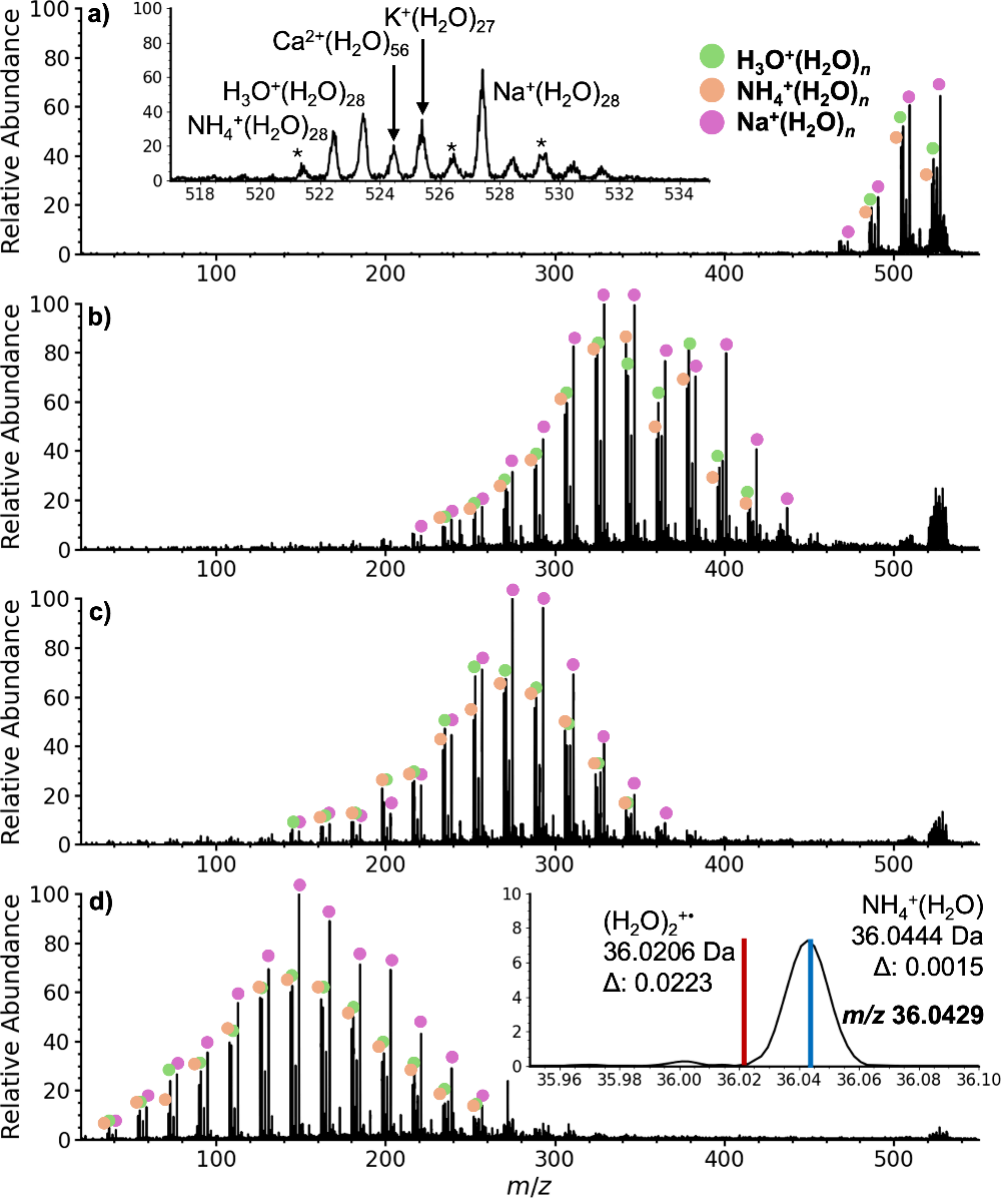
Collision induced dissociation to confirm the identity of the *m*/*z* = 522 ion as NH_4_^+^(H_2_O)_28_ a) precursor isolation (lower mass fragments corresponding to sequential loss of water molecules due to some ion activation that occurs during isolation), and collision voltages of b) 10 V, c) 20, and d) 30 V. The inset in d) shows the accurate mass measurement identifying the *m*/z 36 ion as NH_4_^+^(H_2_O) (blue line) and not (H_2_O)_2_^+•^ (red line), confirming the identity of this entire ion series as NH_4_^+^(H_2_O)_*n*_. Starred peaks correspond to recurring organic contaminants.

To confirm that larger water clusters that incorporate an ammonium ion would produce a *m*/*z* 36 ion under the harsher (more energetic) conditions that are more commonly employed in mass spectrometry, and in many prior experiments where large water clusters are not typically observed, clusters in the region around NH_4_^+^(H_2_O)_28_ were isolated and collisionaly activated (Figure 3). Higher collision energies lead to increasing fragmentation by sequential loss of water molecules, and abundant *m*/*z* 36 ions are produced at 30 V collision energy (Figure 3d). Accurate mass measurements (36.0429 Da, Δ*m* = 0.0015) confirms that this ion is NH_4_^+^(H_2_O) and not (H_2_O)_2_^+•^. Thus, this would be one of the most abundant ions under more commonly employed instrument conditions that are more energetic and are used to produce bare ions without extensive water attachment.

**Figure 4:**
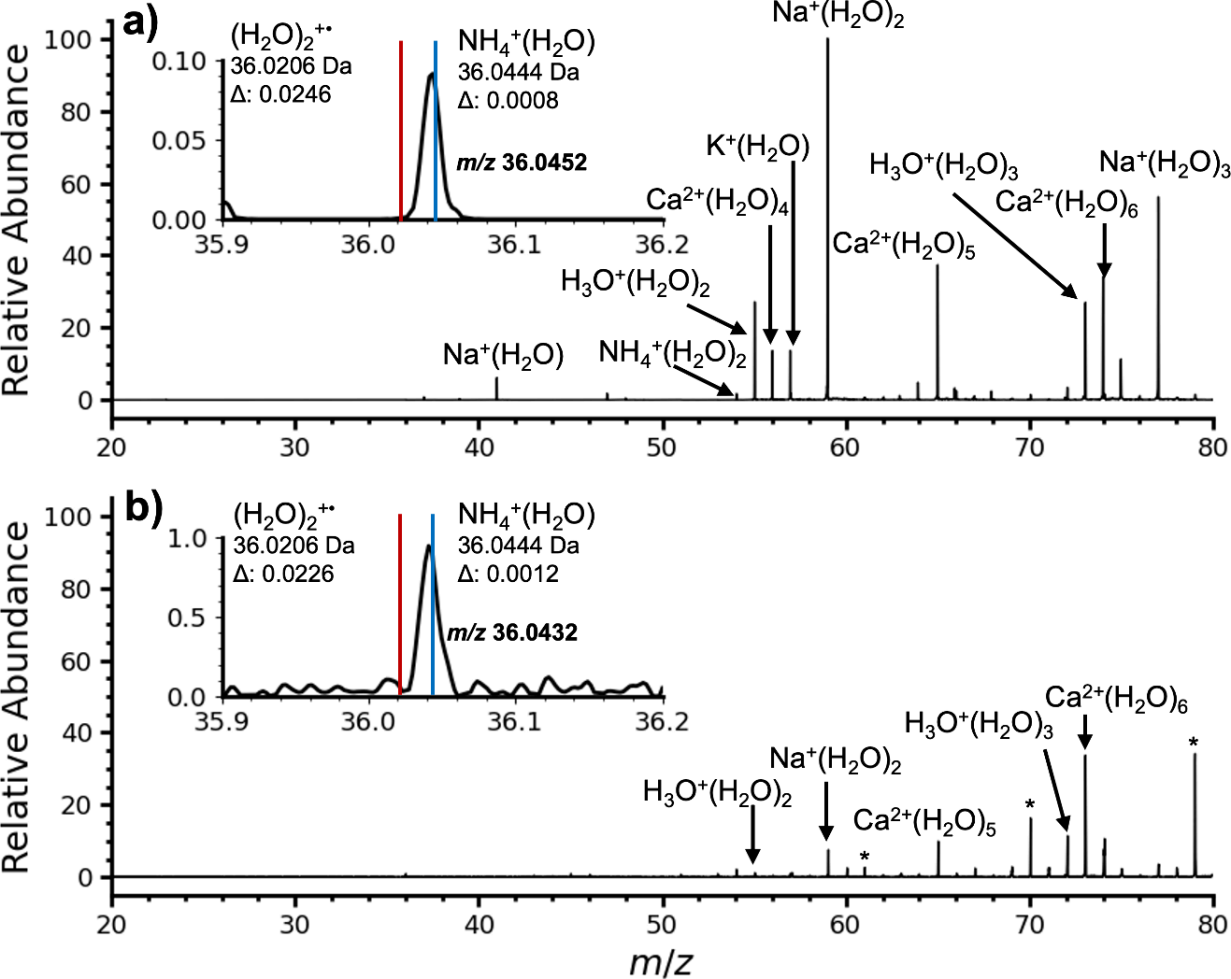
Mass spectra of pure water from a) nanoelectrospray ionization and b) a mesh screen nebulizer. Insets show expansions around *m*/*z* 36 showing the accurate mass measurements and exact masses of NH_4_^+^(H_2_O) (blue lines) and (H_2_O)_2_^+•^ (red lines). Starred peaks indicate contaminants that likely originate from the plastic housing of the mesh screen nebulizer.

With the identity of the *m*/*z* 36 ion and higher order protonated ammonia–water clusters unambiguously determined, along with the likely source of ammonia that leads to the majority of these ions, we investigated whether these same ions are formed from pure water droplets without acid. A nanoelectrospray spectrum of pure water and a spectrum obtained using a vibrating mesh nebulizer without any voltage or pneumatic nebulization applied are shown in Figure 4a and 4b, respectively.

There is a very low abundance of *m*/*z* 36 ions in both spectra. Accurate mass measurements show that these ions are NH_4_^+^(H_2_O) and not (H_2_O)_2_^+•^. Attempts to not exhale breath for the duration of these experiments were unsuccessful, so the background source of the protonated ammonia–water clusters cannot be unambiguously determined from these experiments alone. Purging the source housing with gaseous N_2_ resulted in a 3-fold reduction in the abundance of the *m*/*z* 36 ion with negligible signal remaining, indicating that ammonia in air is by far the dominant source of NH_4_^+^(H_2_O) (Figure S5). The background of this ion in many of these experiments could also be from droplet pickup of ammonium salts commonly used in HPLC separations and native mass spectrometry experiments. Although the source interface was cleaned prior to these experiments, some background contamination may have remained and could lead to trace levels of hydrated ammonium ions that are detected.

The identity of the *m*/*z* 36 ions formed from water droplets has been previously attributed to either (H_2_O–OH_2_)^+•^, (H_3_O + OH)^+•^, or (H_2_O)_2_^+•^, and this ion has been used to support the hypothesis that abundant hydroxyl radicals are formed at the surface of water droplets owing to the high intrinsic electric field^7^ at the surface of even uncharged droplets.^15^ Our results show that this ion has likely been misidentified in prior experiments. Water nanodrops and microdroplets can lead to the production of hydrated ammonium ions at the same nominal mass, but there is no detectable level of an ionized water dimer in this or other experiments in which this ion has been unambiguously identified by accurate mass measurements. While it is possible that ionized water dimer could be formed by electronic excitation that occurs in pneumatic nebulization sources,^21,22,24−26^ or by electron emission from negatively charged droplets and subsequent electron ionization,^25^ the results presented here, as well as other results,^21^ indicate that this ion and abundant hydroxyl radical formation does not occur at the surface of unactivated water droplets with initial diameters between ∼100 nm and 20 μm. These results also show that unexpected ions can be formed in droplet experiments because of exposure to background molecules,^41,42^ even those at trace levels. These factors should be carefully considered before attributing to unusual chemistry to the high intrinsic electric field at the surfaces of water microdroplets. It should be noted that measuring gas-phase ions does not directly probe the nature of intermediates at droplet surfaces, so these results do not rule out the possibility of interesting chemistry that might occur at droplet surfaces as a result of the unique nature of the interface. For example, electron emission from negatively charged aqueous droplets that are charged near the Rayleigh limit likely lead to the formation of hydroxyl radicals at the droplet surface,^25^ and analyte concentration that occurs due to droplet evaporation^5^ may lead to reactive chemistry. However, careful controls that can rule out other factors involved in unusual chemistry in microdroplets, such as environmental ozone^19^ and ammonia,^21^ dissolved oxygen,^18^ red-ox chemistry in solution^18,43^ or in the gas phase,^25^ and electronic excitation^25^ are necessary.

The abundance of protonated ammonium clusters in these experiments indicates an unoptimized detection limit for gaseous ammonia in charged microdroplets in the low parts-per-billion range (Figure S5). Ammonia concentration is typically higher in indoor air (∼10–70 ppb) than in outdoor air (∼50 ppt to 5 ppb),^44^ but can be substantially higher near agriculture and industrial sources. Surfaces have been shown to serve as large reservoirs of gaseous ammonia.^45^ Dermal emissions of ammonia can be substantially higher than breath emissions,^46^ and may also be a likely source of hydrated ammonium in these and other experiments. The likely misidentification of baseline *m*/*z* 36 ion as (H_2_O)_2_^+•^ in many prior droplet experiments, as well as the high sensitivity of the mass spectrometers to trace levels of gaseous ammonia in these experiments, suggests that the evidence for production of abundant ammonia from ambient nitrogen gas in charged microdroplets experiments at room temperature^9,10^ may also require more critical evaluation.

## Methods

Experiments were performed using a Waters Q-TOF Premier mass spectrometer (Milford, MA) using a sample cone, extraction cone, and ion guide potentials of 70.0, 2.0, and 2.0 V, respectively, and a source block temperature of 50 °C. No gas was introduced into the instrument for these experiments. Isolation of the precursor was done with a collision voltage set to 2.0 V, and no collision gas was introduced into the instrument. The low and high mass resolutions of the quadrupole were set to 4.7 and 15.0, respectively, in order to transmit a range of clusters in the *m*/*z* ∼ 523 region. For collision induced dissociation experiments, argon gas was introduced into the instrument at a flow rate of 0.1 mL/min. Borosilicate capillaries (1.5 mm outer diameter, Sutter Instruments, Novato, CA) were pulled to tip diameters of 1.7 μm with a Flaming/Brown P-87 micropipet puller (Sutter Instruments, Novato, CA). The capillaries were positioned ∼3 mm from the mass spectrometer sampling cone. Electrospray was initiated by applying +0.7 – + 1.0 kV to a platinum wire that is in contact with the solution in the emitters. For experiments with the mesh screen nebulizer, the device was placed approximately 15 cm away from the instrument inlet, and data were acquired for 20 min. The nebulizer runs on battery power; no external voltage is applied to the nebulizer or the solution contained in the nebulizer. The instrument was externally calibrated using a 2 μg/mL solution of sodium iodide in 50/50 isopropanol/water, and the (Na_*n*_I_*n*–1_)^+^ ion series was used to calibrate the instrument. Each spectrum was recalibrated using sodium, potassium, and hydrated calcium ions as internal standards using MassLynx v. 4.1. Water from a Milli-Q gradient ultrapure water purification system (Millipore, Billerica, MA) was used. For the breath exhalation experiments, a person who was not the instrument operator exhaled one breath ∼ 12 in. from the housing of the mass spectrometer inlet or directly into the enclosure (Figure S7). In separate experiments, four individuals each exhaled one breath approximately 12 in. from the housing (Figure S7). In each of these experiments, the individuals consumed various amounts and types of protein and other food items ∼ 3 – 5 h prior to the experiments. No unexpected or unusually high safety hazards were encountered.

## Supporting Information Available

Experimental setup of photographs, data for LaCl_3_, exhalation of breath adjacent to source housing, and from four individuals’ exhalation near the instrument, total ion current plots, tabulations of exact mass measurements, and relative abundances of *m*/*z* 36: *m*/*z* 37 (PDF)
