# Supplementary material for: A Source of the Mysterious m/z 36 Ions Identified: Implications for the Stability of Water and Unusual Chemistry in Microdroplets
Source: ACS Cent Sci. 2025 Apr 4;11(4):622–8. doi: 10.1021/acscentsci.5c00306 (PMC12022912; doi:10.1021/acscentsci.5c00306)
Supplement: Supplementary file 1 — oc5c00306_si_001.pdf [file oc5c00306_si_001.pdf]

Supporting information for

A Source of the Mysterious *m/z* 36 Ions Identified: Implications for the Stability of Water and Unusual Chemistry in Microdroplets

Casey J. Chen and Evan R. Williams*

Department of Chemistry, University of California, Berkeley, CA 94720 USA

*To whom correspondence should be addressed

Email: erw@berkeley.edu

Table of Contents

Figure S1. Nanoelectrospray mass spectrum of an aqueous 10 μM $LaCl_3$ solution.

Table S1. Relative abundances of ammonia and water clusters before and after exhalation of a single breath

Figure S2. Total ion current from nanoelectrospray of a 1.0 μM aqueous acetic acid solution as a function of time and exhalation of a single breath adjacent to the source housing

Figure S3. Mass spectrum acquired during the exhalation of breath adjacent to source housing in Figure S1

Table S2. Accurate mass measurements and exact mass of various ions produced by nanoelectrospray from a 1.0 μM aqueous acetic acid solution.

Table S3. Potential elemental compositions of the ion at *m/z* 36

Figure S4. Total ion current from nanoelectrospray of a 1.0 μM aqueous acetic acid solution as a function of time and mass spectra acquired from four individuals exhaling a single breath ~12 inches

Figure S5. Mass spectrum of pure water from nanoelectrospray with the source enclosed before and after breathing, and after purging from N_2 gas

Figure S6. Photos of the experimental set up used in the N_2 source purge experiments.

Figure S7. Photos of the experimental set up with indication of where exhalation occurred

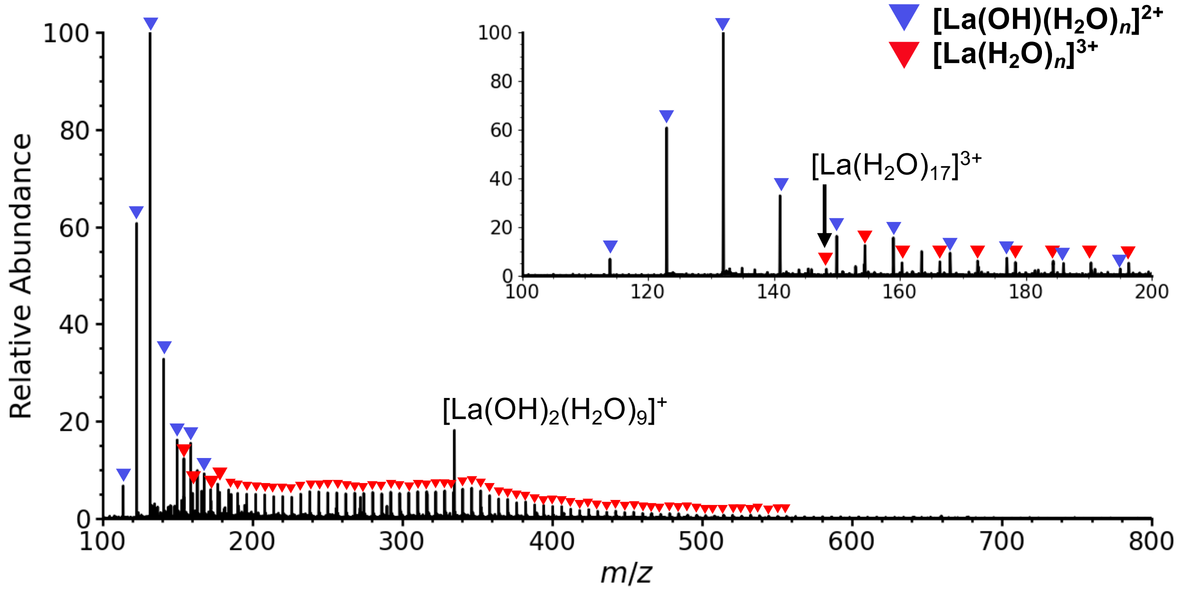

Figure S1. Nanoelectrospray mass spectrum of a 10 µM aqueous $LaCl_3$ solution. Blue triangles indicate the series for $[La(OH)(H_2O)_n]^{2+}$, red triangles indicate the series for $[La(H_2O)_n]^{3+}$. Inset is an expansion around the region between *m/z* 100 and 200. The smallest cluster containing La^{3+} has 17 water molecules with up to $n = 90$ observed for $[La(H_2O)_n]^{3+}$.

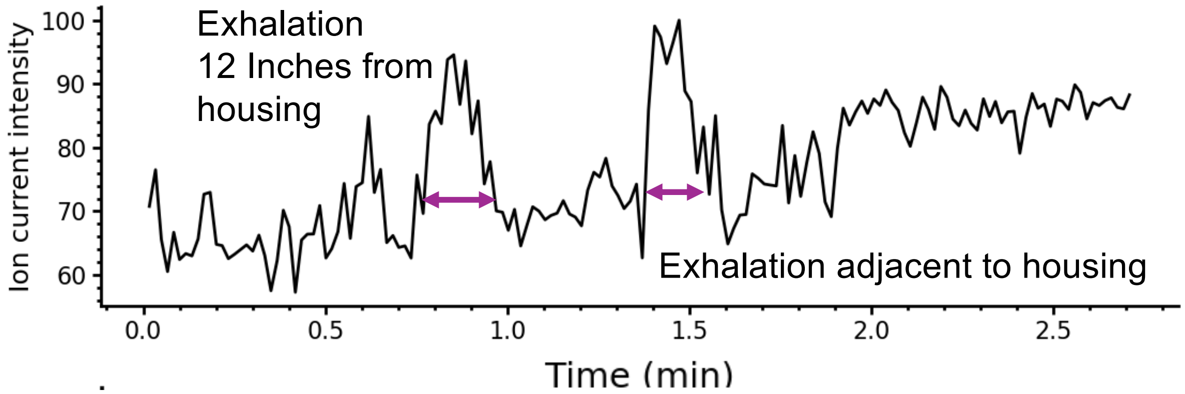

Figure S2. The total ion current as a function of time from nanoelectrospray of a 1.0 μM aqueous acetic acid solution. Sections when a single breath was exhaled approximately 12 inches from the instrument source housing and when the exhalation occurred adjacent to the source housing are marked on the figure (Figure S7).

**Number of water molecules (*n*)**	**Relative abundance of $NH_4^+(H_2O)_n$:$H3O^+(H_2O)_n$**		
	**Without exhaled breath**	**Exhaled breath ~12 inches from housing**	**Exhaled breath next to housing**
1	0.12	1.80	3.52
2	0.11	1.27	2.26
3	0.15	1.75	2.64
4	0.31	3.31	5.07
5	0.34	3.51	4.93
10	0.40	3.75	5.18
15	0.42	3.57	4.67
20	0.46	3.91	4.79
25	0.35	4.07	4.88

Table S1. Abundances of hydrated protonated ammonia clusters relative to protonated water clusters for ions at select cluster size formed by nanoelectrospray of a 1.0 µM aqueous acetic acid solution.

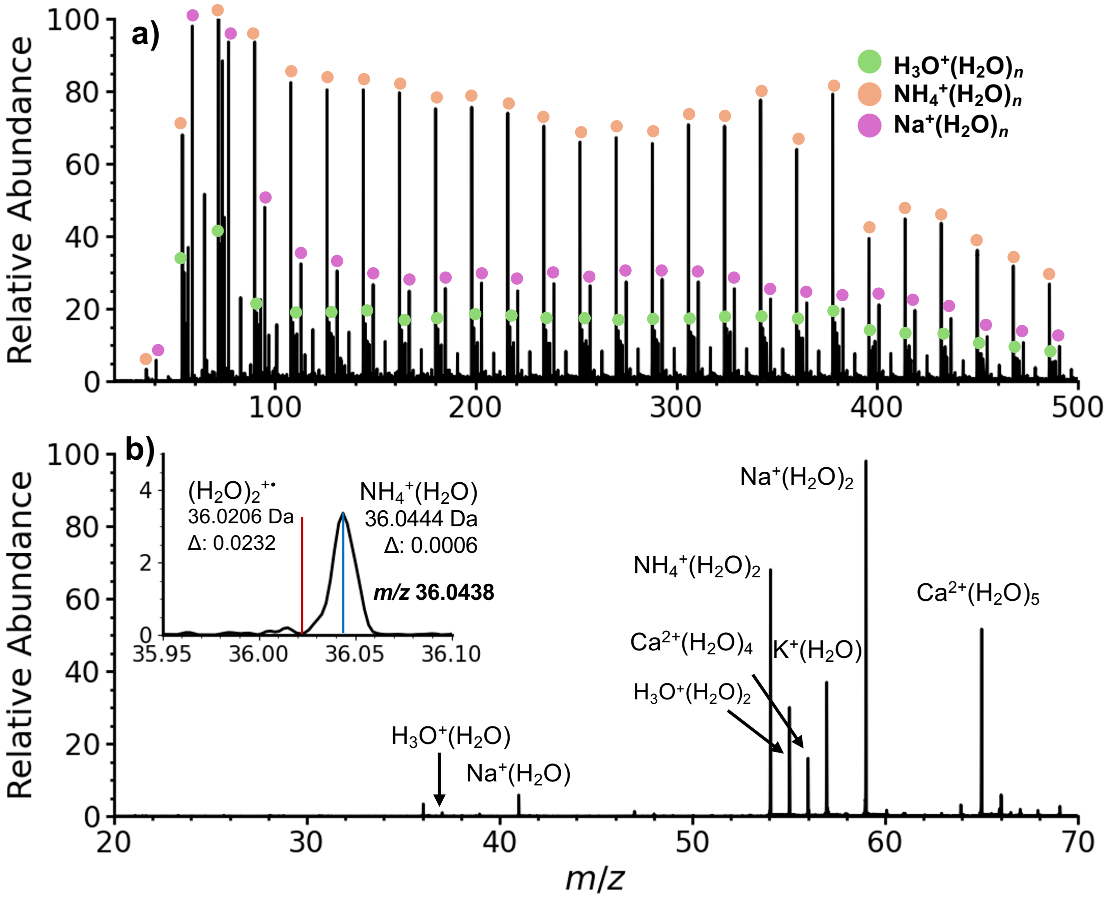

Figure S3. Nanoelectrospray mass spectrum of a 1.0 μM aqueous acetic acid solution in the region from a) m/z = 20 – 500 and b) m/z = 20 – 70 when a single breath was exhaled directly adjacent to the instrument source housing (Figure S7). The inset in b) is an expansion around m/z = 36 showing that the accurate measured mass is consistent with $NH_4^+(H_2O)$ and not $(H_2O)_2^{+\bullet}$

Sample	Species	Exact Mass (Da)	Measured Mass (Da)	Mass Difference (Da)
1.0 µM aqueous acetic acid aqueous solution, no exhalation, nESI	Na^+	22.9892	22.9897	0.0005
	$NH_4^+(H_2O)$	36.0444	36.0438	0.0006
	$(H_2O)_2^{+\bullet}$	36.0206	36.0438	0.0232
	$H_3O^+(H_2O)$	37.0284	37.0274	0.0010
	K^+	38.9632	38.9629	0.0003
	$Na^+(H_2O)$	40.9998	40.9996	0.0002
	$NH_4^+(H_2O)_2$	54.0550	54.0561	0.0011
	$H_3O^+(H_2O)_2$	55.0389	55.0378	0.0012
	$Na^+(H_2O)_2$	59.0094	59.0094	0.0010
1.0 µM aqueous acetic acid aqueous solution, exhalation 12 ft from housing, nESI	Na^+	22.9892	22.9876	0.0005
	$NH_4^+(H_2O)$	36.0444	36.0438	0.0006
	$(H_2O)_2^{+\bullet}$	36.0206	36.0438	0.0232
	$H_3O^+(H_2O)$	37.0284	37.0274	0.0010
	K^+	38.9632	38.9629	0.0003
	$Na^+(H_2O)$	40.9998	40.9996	0.0002
	$NH_4^+(H_2O)_2$	54.0550	54.0564	0.0014
	$H_3O^+(H_2O)_2$	55.0389	55.0378	0.0011
	$Na^+(H_2O)_2$	59.0094	59.0103	0.0009
Water, no exhalation, nESI	Na^+	22.9892	22.9897	0.0005
	$NH_4^+(H_2O)$	36.0444	36.0452	0.0008
	$(H_2O)_2^{+\bullet}$	36.0206	36.0452	0.0246
	$H_3O^+(H_2O)$	37.0284	37.0273	0.0011
	K^+	38.9632	38.9641	0.0009
	$Na^+(H_2O)$	40.9998	40.9993	0.0005
	$NH_4^+(H_2O)_2$	54.0550	54.0549	0.0019
	$H_3O^+(H_2O)_2$	55.0389	55.0398	0.0011
	$Na^+(H_2O)_2$	59.0094	59.0115	0.0021
Water, nebulizer	Na^+	22.9892	22.9894	0.0002
	$NH_4^+(H_2O)$	36.0444	36.0432	0.0012
	$(H_2O)_2^{+\bullet}$	36.0206	36.0432	0.0226
	$Na^+(H_2O)$	40.9998	41.0005	0.0007
Water, $H_3O^+(H_2O)_{28}$ cluster activated with 35V collisional activation, nESI	Na^+	22.9892	22.9910	0.0018
	$NH_4^+(H_2O)$	36.0444	36.0429	0.0015
	$(H_2O)_2^{+\bullet}$	36.0206	36.0429	0.0223
	$H_3O^+(H_2O)$	37.0284	37.0285	0.0001
	K^+	38.9632	38.9638	0.0006
	$Na^+(H_2O)$	40.9998	41.0002	0.0004
	$NH_4^+(H_2O)_2$	54.0550	54.0561	0.0011
	$H_3O^+(H_2O)_2$	55.0389	55.0375	0.0014
	$Na^+(H_2O)_2$	59.0094	59.0119	0.0015

Table S2. Accurate mass measurements and exact masses of various ions produced by either nanoelectrospray ionization (nESI) or a mesh screen nebulizer using a Waters Q-TOF Premier mass spectrometer.

Elemental composition	Exact Mass (Da)	Mass difference from *m/z* 36.0438 (Da)
$C_3^{+\bullet}$	35.9995	0.0443
$NH_4^+(H_2O)$	36.0444	0.0006
$(H_2O)_2^{+\bullet}$	36.0206	0.0232
$SH_4^{+\bullet}$	36.0028	0.0410

Table S3. Elemental compositions for *m/z* 36 and the mass difference from *m/z* 36.0438 (measured from a 1.0 μM aqueous acetic acid aqueous solution)

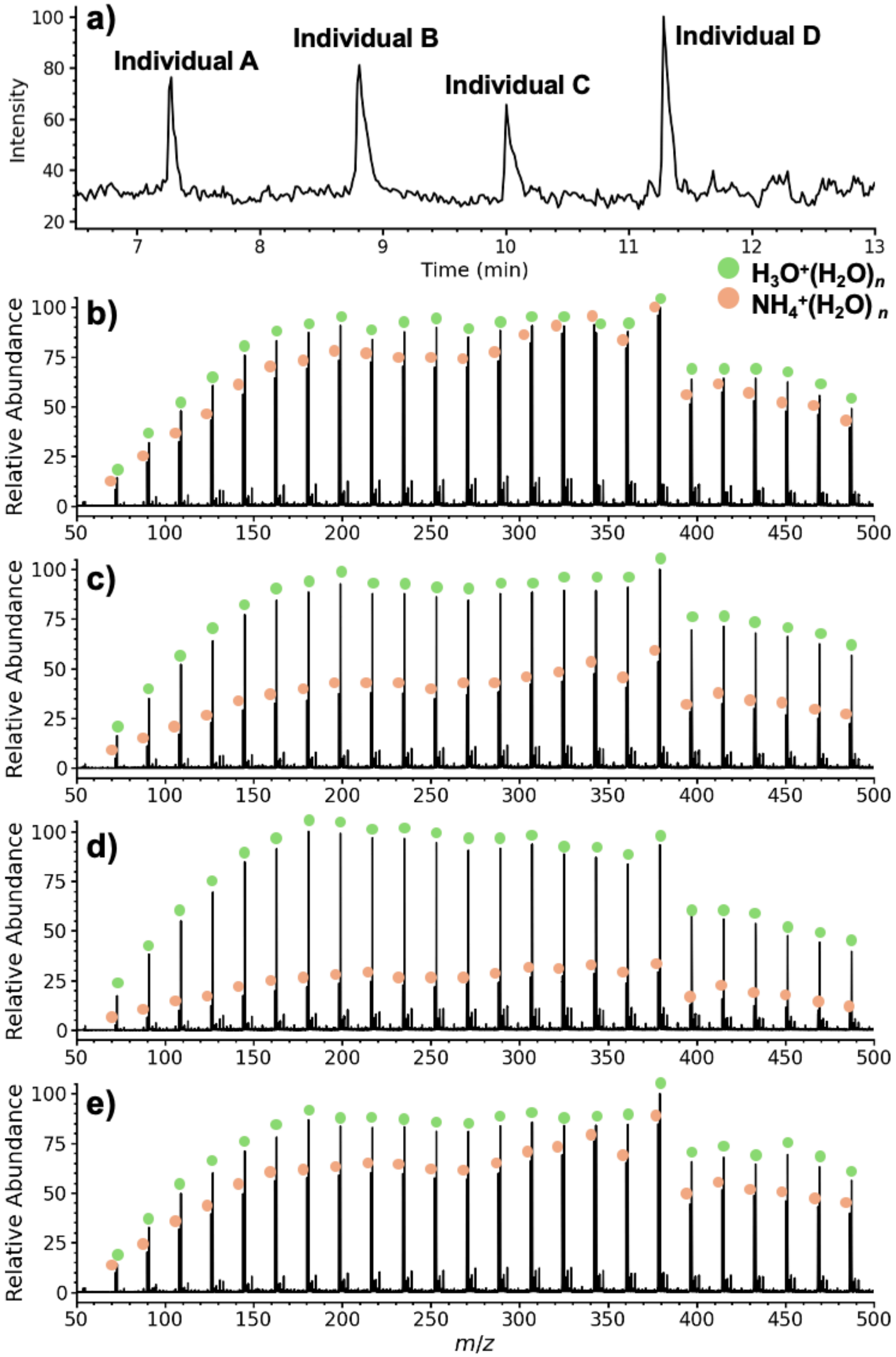

Figure S4. Results from nanoelectrospray ionization of a 1.0 μM aqueous acetic acid solution when four different individuals exhaled 12 inches from the mass spectrometer source housing a) total ion current as a function of time showing peaks when each of the four individuals exhaled a single breath, and b) through e) mass spectra for each of the individuals upon exhaling.

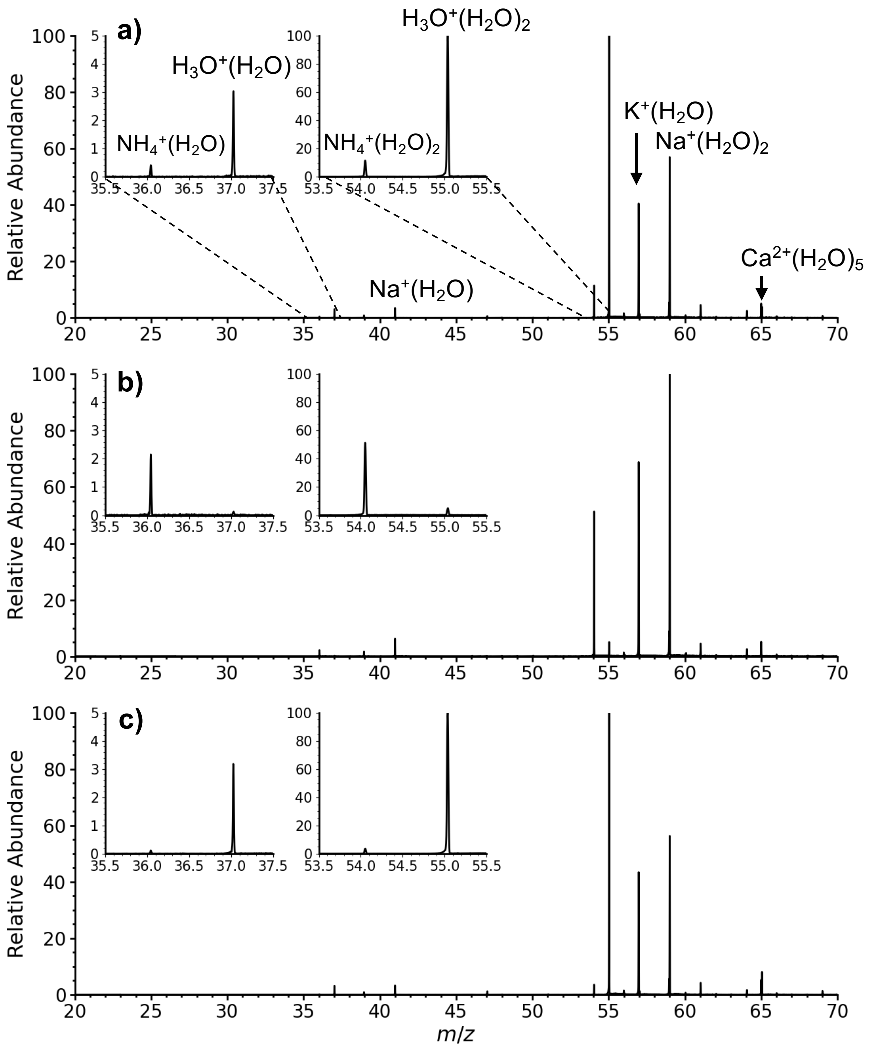

Figure S5. Nanoelectrospray mass spectrum from pure water when a) the sourcing is enclosed, b) breath is exhaled into the enclosed source, and c) after 15 minutes of purging the source with N_2 gas (Figure S6). Insets show expansions around *m*/*z* = 35.5 – 37.5 and 53.5 – 55.5. The S/N for $NH_4^+(H_2O)$ and $NH_4^+(H_2O)_2$ in b) is 50 and 650, respectively. Based on the average ammonia concentration exhaled through breath in a healthy individual (265 ppb) and the S/N of these ions, the approximate and unoptimized lower limit of detection of gaseous ammonia using these ions is ~16 ppb and ~1.2 ppb, respectively (S/N = 3 for lower limit of detection).

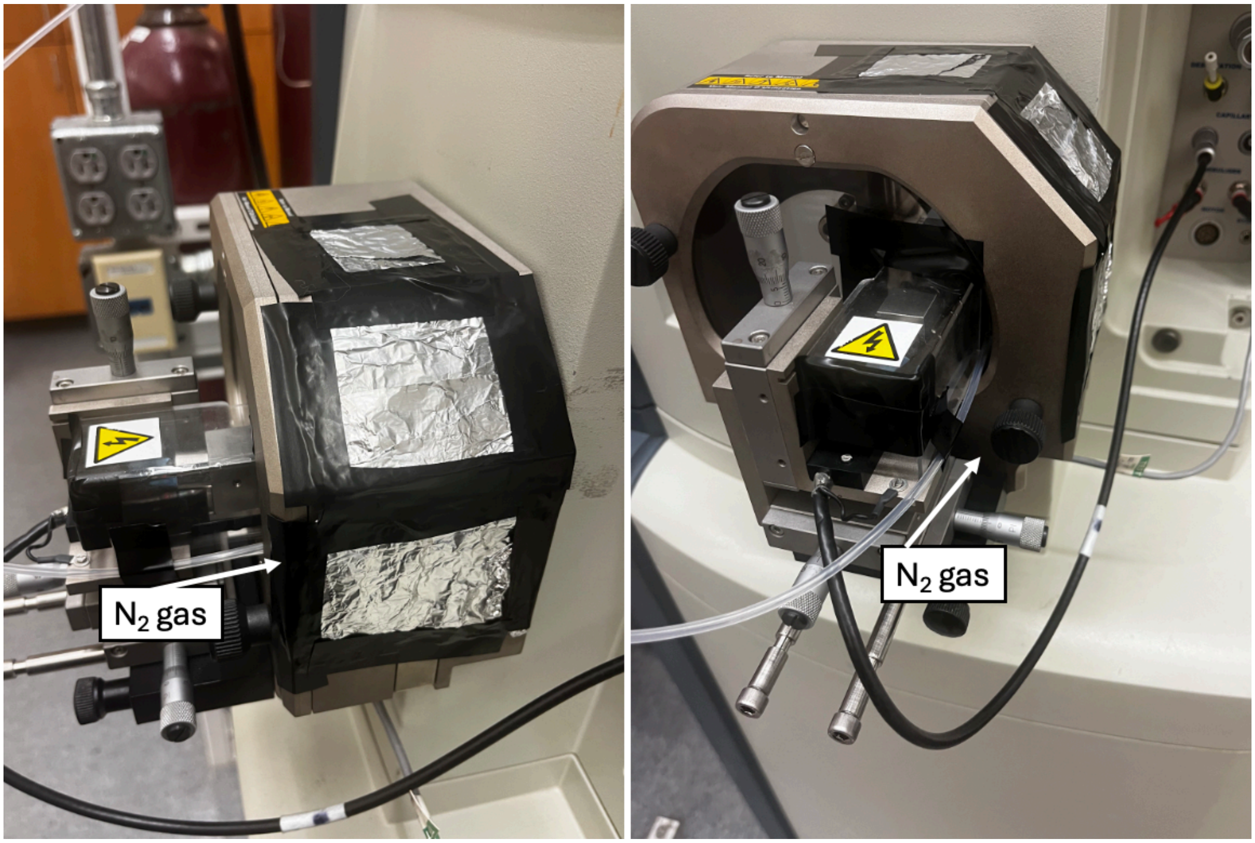

Figure S6. Image of source housing enclosed with foil; arrow indicates the flow of N_2 gas. The source housing was purged for 15 minutes with N_2 gas before acquiring the spectrum shown in Figure S5c.

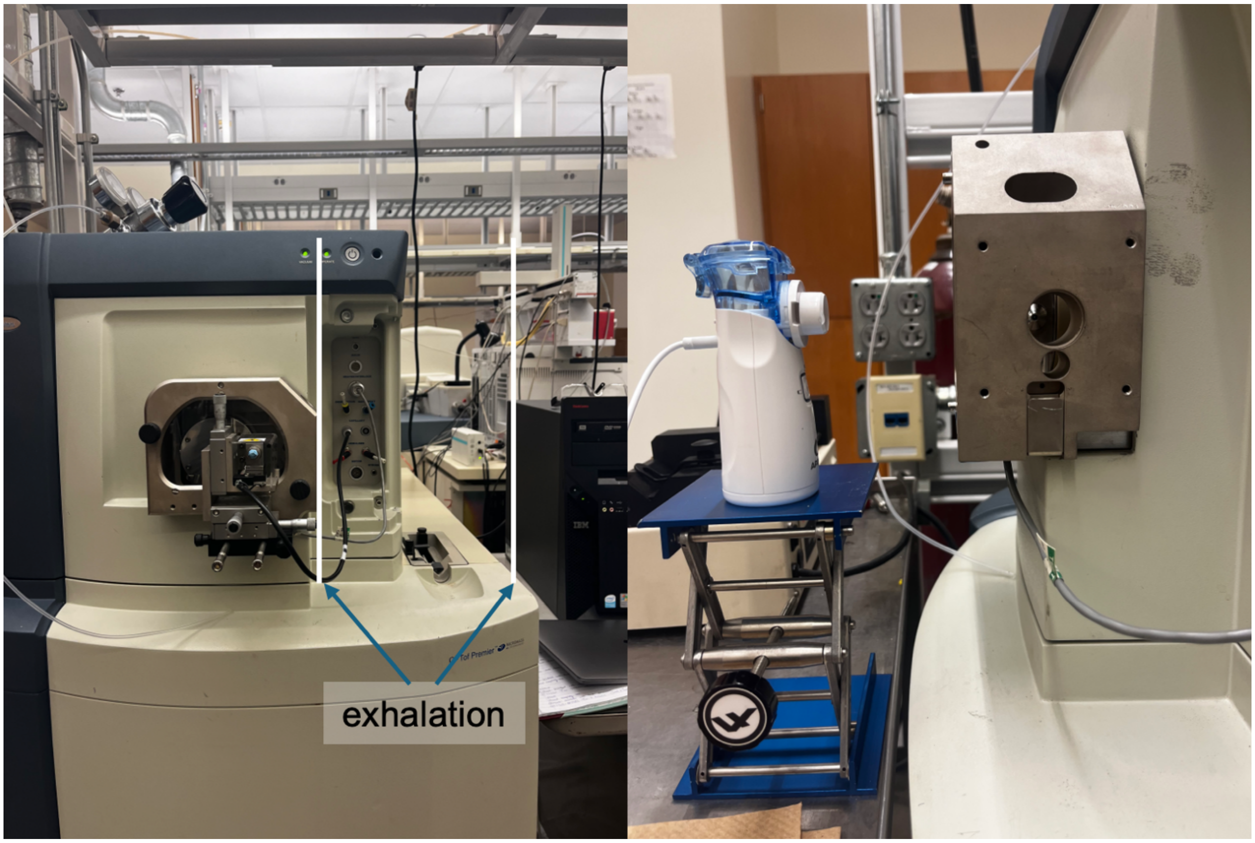

Figure S7. Pictures of the experimental set up, white lines indicate where the breath exhalation occurred.
